# Prognostic Value of Bone Marrow Uptake Using 18F-FDG PET/CT Scans in Solid Neoplasms

**DOI:** 10.3390/jimaging8110297

**Published:** 2022-10-31

**Authors:** Francisco Tustumi, David Gutiérrez Albenda, Fernando Simionato Perrotta, Rubens Antonio Aissar Sallum, Ulysses Ribeiro Junior, Carlos Alberto Buchpiguel, Paulo Schiavom Duarte

**Affiliations:** 1 Department of Gastroenterology, Digestive Surgery Division, Universidade de São Paulo, Av. Dr. Eneas de Carvalho Aguiar 255, São Paulo 05403-000, SP, Brazil; 2 Department of Radiology and Oncology, Nuclear Medicine Division, Universidade de São Paulo, Av. Dr. Eneas de Carvalho Aguiar 255, São Paulo 05403-000, SP, Brazil

**Keywords:** bone marrow, esophageal neoplasms, neoadjuvant therapy, positron emission tomography, nuclear medicine

## Abstract

Background: Fluorine-18-fluorodeoxyglucose positron emission tomography/computerized tomography (18F-FDG PET/CT) uptake is known to increase in infective and inflammatory conditions. Systemic inflammation plays a role in oncologic prognosis. Consequently, bone marrow increased uptake in oncology patients could potentially depict the systemic cancer burden. Methods: A single institute cohort analysis and a systematic review were performed, evaluating the prognostic role of 18F-FDG uptake in the bone marrow in solid neoplasms before treatment. The cohort included 113 esophageal cancer patients (adenocarcinoma or squamous cell carcinoma). The systematic review was based on 18 studies evaluating solid neoplasms, including gynecological, lung, pleura, breast, pancreas, head and neck, esophagus, stomach, colorectal, and anus. Results: Bone marrow 18F-FDG uptake in esophageal cancer was not correlated with staging, pathological response, and survival. High bone marrow uptake was related to advanced staging in colorectal, head and neck, and breast cancer, but not in lung cancer. Bone marrow 18F-FDG uptake was significantly associated with survival rates for lung, head and neck, breast, gastric, colorectal, pancreatic, and gynecological neoplasms but was not significantly associated with survival in pediatric neuroblastoma and esophageal cancer. Conclusion: 18F-FDG bone marrow uptake in PET/CT has prognostic value in several solid neoplasms, including lung, gastric, colorectal, head and neck, breast, pancreas, and gynecological cancers. However, future studies are still needed to define the role of bone marrow role in cancer prognostication.

## 1. Introduction

The fluorine-18-fluorodeoxyglucose positron emission tomography/computerized tomography (18F-FDG PET/CT) is a functional test routinely used for staging in many cancers, including esophageal, gynecological, melanoma, and head and neck [1]. This test is usually performed to detect lymph nodal and distant metastasis, as well as for prognostic estimation and evaluation of tumor response [1,2]. 18F-FDG PET/CT is also extensively applied in hematological malignancy evaluation, such as lymphoma (Hodgkin’s and Non-Hodgkin’s), multiple myeloma, leukemia, and solitary plasmocytoma. FDG high uptake in nodal, spleen, liver, or bone marrow usually suggests cancer aggressiveness behavior [3].

However, certain findings may contribute to false-positive FDG uptake. PET/CT may show high uptake when there is an infection or inflammation in the course [4]. As a result, the use of PET/CT has expanded, and it now has an established role in a number of infection and inflammatory conditions [4,5]. 18F-FDG may have increased uptake in infection sites and in the immune organs during active inflammation, such as the bone marrow and spleen [6].

Cancer may induce systemic changes in the immune system. The immunologic changes and the inflammatory status in cancer patients contribute to malignant cell elimination [7]. Several cytokines released by immune cells or secreted by tumor cells can influence cancer growth rate or dissemination [8]. Consequently, patients with advanced cancer have increased inflammation associated with biomarkers, such as C-reactive protein, red cell distribution width (RDW), platelet-to-lymphocyte ratio, and the neutrophil-to-lymphocyte ratio [9,10,11,12,13,14,15].

Stress and systemic inflammation enhance bone marrow metabolism, with bone marrow mesenchymal stem cells activation and differentiation [16,17,18]. Aerobic glycolysis is the major energy that promotes the differentiation of bone marrow mesenchymal stem cells [19]. Considering that 18F-FDG uptake by tissues is a marker of glucose metabolism, bone marrow increased uptake in oncology patients could potentially depict the systemic cancer burden. Thus, this study aimed to evaluate the prognostic value of 18F-FDG uptake in the bone marrow before cancer treatment.

## 2. Materials and Methods

The present study combines an institutional cohort evaluation and a systematic review of the literature, evaluating the prognostic value of 18F-FDG uptake in the bone marrow of patients imaged with PET/CT prior to cancer therapy. The systematic review evaluates solid neoplasms and treatments. The review included gynecological, lung, pleura, breast, pancreatic, head and neck, esophageal, stomach, colorectal, and anal cancer. The single institute cohort evaluates 18F-FDG PET/CT scans from patients with esophageal cancer undergoing trimodal therapy.

### 2.1. Cohort Analysis

#### 2.1.1. Study Design

Consecutive patients from 2009 to 2019 of a retrospective cohort study were evaluated in a group of esophageal cancer patients. The local ethics committee approved the study.

#### 2.1.2. Eligibility Criteria

Patients with esophageal carcinoma (adenocarcinoma or squamous cell carcinoma) submitted to 18F–FDG PET/CT imaging prior to neoadjuvant platinum and taxane chemotherapy associated with radiotherapy, followed by esophagectomy, were included.

Patients with any clinical suspicion of active or recent infection or that used any drug to stimulate leukocytes were excluded. Patients with distant metastases were also excluded from the study.

#### 2.1.3. Preoperative Workup

All patients were evaluated with 18F–FDG PET/CT and endoscopy. Esophageal neoplasms were staged according to the current edition of the Union for International Cancer Control (UICC) staging system [20].

#### 2.1.4. PET/CT

Images were acquired on a Discovery 690 PET/CT scanner with time-of-flight (General Electric, Waukesha, WI, USA). The patients fasted for six hours, and their blood glucose level was kept below 180 mg/dl before the 18F-FDG injection (3.7 MBq of 18F-FDG/kg). Imaging was initiated 1 h after the injection. A single highly experienced nuclear medicine physician (P.S.D.), blinded to the clinical data, using AW VolumeShare 5 workstation (General Electric, Waukesha, WI, USA), evaluated the PET/CT tests.

**Figure 1:**
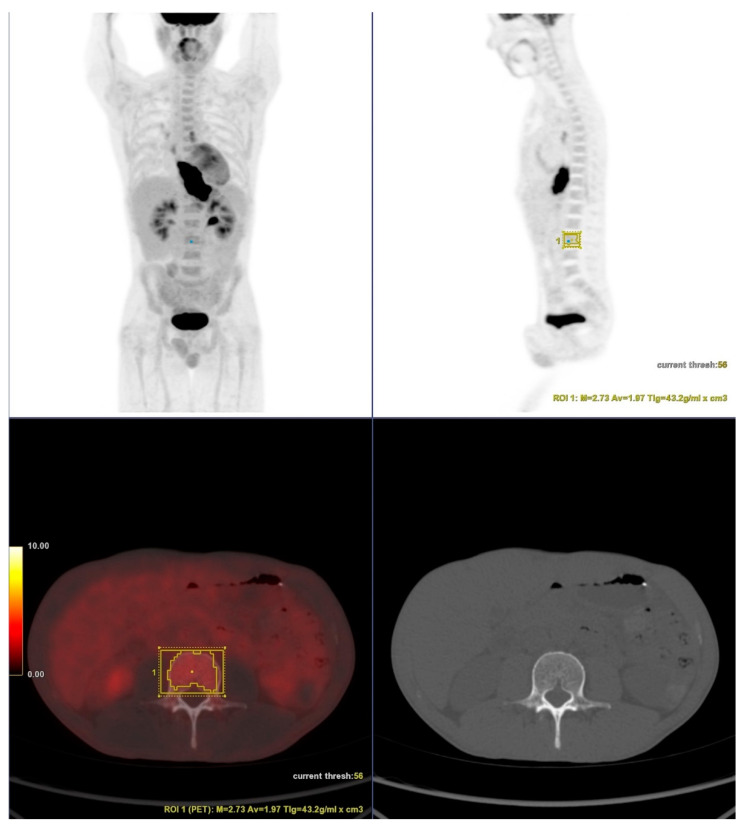
18F-FDG PET/CT showing a patient with esophageal cancer. Volumes of interest (VOIs) were drawn over in one of the L3-L5 vertebral bodies, preferably L3. Care was taken to exclude bones with fractures, as shown in CT slice. Automatic isocontour was generated, and the mean SUV was calculated.

Volumes of interest (VOIs) were drawn over in one of the L3-L5 vertebral bodies, preferably L3 (Figure 1). Care was taken to exclude bones with fractures. Automatic isocontour (set at 75% of the maximum SUV) was generated, and the mean SUV was calculated.

The bone marrow-to-liver SUVmean ratio (BML) was calculated by dividing bone SUVmean marrow to liver SUVmean. The liver mean SUV was extracted by drawing a 1 cm diameter circle of region of interest (ROI) over different points in liver images from the 18F-FDG PET/CT (5-10 ROIs).

#### 2.1.5. Outcomes

The outcomes investigated were disease-free survival (DFS), pathological complete response to neoadjuvant therapy (defined as the absence of malignant cells in the surgical specimen), and oncologic clinical stage (cStage).

#### 2.1.6. Statistical Analyses

The categorical variables were described as absolute or relative frequencies, and any associations were verified with the chi-square test, Fisher’s exact test, or likelihood ratio test. Continuous variables were assessed with the analysis of variance (ANOVA) test. The continuous values of BML were converted into binary outcomes according to their median value. Hazard ratio (HR) or odds ratio (OR) was used to measure association. Log-rank test and Kaplan–Meier curves were applied to determine the equality of survivor functions.

Cox proportional hazard regression was used for survival analyses. Logistic regression was used to evaluate associations between the independent variables and pathological responses to neoadjuvant therapy. A univariate analysis was performed to assess the association of the independent variables with overall survival. The variables with a statistically significant association with survival in the univariate analyses (*p* < 0.05) were selected for multivariate analyses.

The data were analyzed with the STATA 16.1 Release 16 software (StataCorp LLC, College Station, TX, USA). A significance level of *p* < 0.05 was adopted for statistical significance.

### 2.2. Systematic Review Analysis

#### 2.2.1. Protocol Register

This systematic review was submitted for the International Prospective Register of Systematic Reviews under the registry CRD42021292482. The systematic review construction was based on the PRISMA guidelines [21].

#### 2.2.2. Search and Selection

Two authors (F.T. and R.A.A.S.) independently searched the following scientific databases: PubMed, Embase, Cochrane, Lilacs, and a manual search for relevant references. The last search was completed in December 2021. The search strategy included a combination of keywords and MeSH terms: “positron emission tomography” OR “PET” OR “PET-CT” OR “PET CT” OR “PET/CT” AND “FDG” OR “18-F-FDG” OR “18F-FDG” OR “18FFDG” OR “18FDG” OR “fluorodeoxyglucose” AND “bone marrow”.

#### 2.2.3. Eligibility

The eligibility criteria were: (1) Studies that evaluate solid neoplasms; (2) Studies evaluating bone marrow 18F-FDG uptake; (3) Articles that investigate any prognostic outcome, such as pathological response and survival rates. Both observational and clinical designs were accepted. Hematological malignancy studies were excluded.

#### 2.2.4. Data Extraction

Two authors (F.T. and R.A.A.S.) independently extracted the data. The following information was extracted: (1) Authors, year of publication, study design; (2) Age, sex, follow-up, cancer type; (3) Therapy; (4) Variables related to the population and outcomes: sample size; survival rates; and pathological response the neoadjuvant therapy.

#### 2.2.5. Risk of Bias and Certainty Assessment

The articles were assessed for bias risk using the ROBINS-I assessment tool [22]. The ROBINS-I tool assesses non-randomized researchers risk of bias, based on the domains confounding, selection, classification of interventions, deviation from intended intervention, missing data, measurement of endpoints, and reporting bias. Grading of Recommendations, Assessment, Development, and Evaluations-GRADE [23] was used for the certainty assessment of the evidence and the strength of recommendations. This tool is based on risk of bias, inconsistency, indirectness, and imprecision.

#### 2.2.6. Synthesis

Considering that the inclusion criteria encompassed several different neoplasms and treatments, the studies presented high clinical heterogeneity. Thus, performing a meta-analysis was not possible, and a qualitative synthesis was undertaken instead.

## 3. Results

### 3.1. Cohort

The cohort included 113 esophageal cancer patients (30 females and 83 males). There were 78 squamous cell carcinoma (SCC) and 35 adenocarcinoma cases. The mean follow-up was 26 months (±25). All patients were submitted to neoadjuvant chemoradiotherapy followed by esophagectomy. The baseline characteristics of the cohort study are reported in Table 1.

#### 3.1.1. Bone Marrow 18-F-FDG Uptake and Pretreatment Clinical Stage

**Figure 2:**
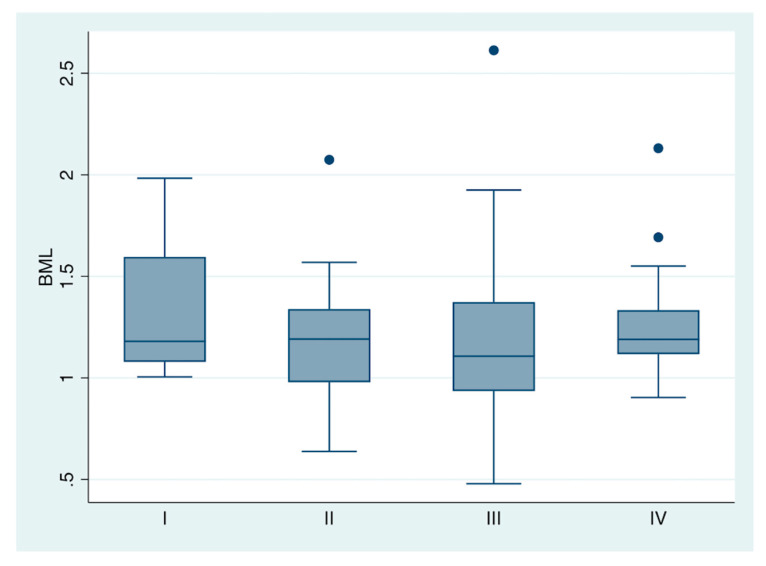
Box plot for esophageal cancer. Bone marrow-to-liver SUV (BML) vs. pretreatment clinical stage.

The BML was not associated with cStage in esophageal cancer treated with trimodal therapy (see Figure 2). The analysis of variance of the four oncologic stages (I, II, III, and IV) demonstrated similar bone marrow uptake between groups (F = 0.59; Prob > F: 0.62).

#### 3.1.2. Serum Laboratory Parameters

**Figure 3:**
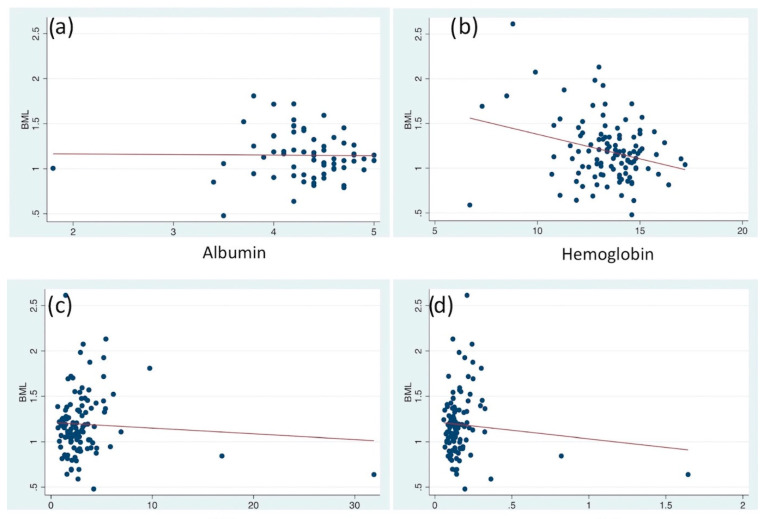
Correlation analysis. Bone marrow-to-liver SUV (BML) was compared with serum albumin (**a**), hemoglobin level (**b**), neutrophil-to-lymphocyte ratio (NLR) (**c**), and platelet-to-lymphocyte ratio (PLR) (**d**).

No significant correlation was noted between BML and pretreatment serum albumin, hemoglobin, NLR, and PLR in esophageal cancer (*p* > 0.05). See Table 2 and Figure 3.

#### 3.1.3. Disease-Free Survival

**Figure 4:**
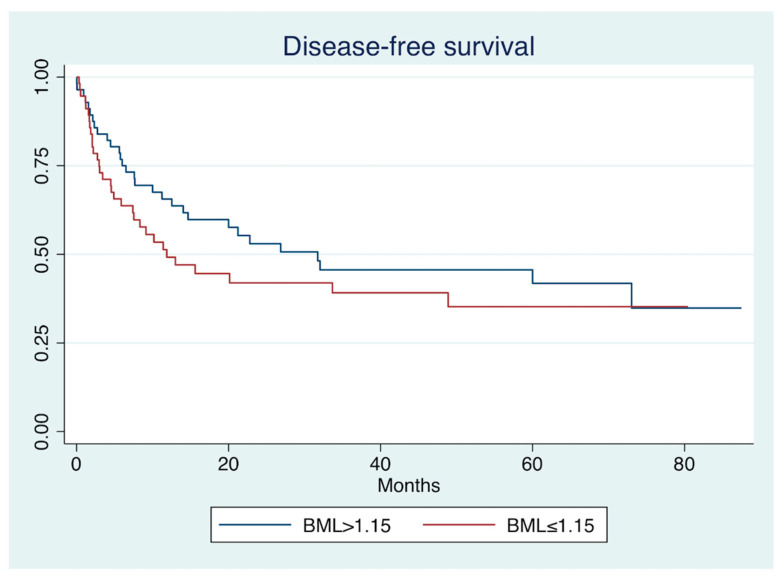
Kaplan–Meier analysis of disease-free survival. Patients were grouped in low bone marrow-to-liver SUV (BML ≤ 1.15) and high BML (>1.15).

A log-rank test for equality of survivor functions found a non-significant difference for DFS between low and high BML (*p* = 0.25). See Figure 4.

In Cox regression, the poor prognostic covariables were advanced age (>65 years old), advanced pretreatment clinical stage (III/IV), adenocarcinoma histology, and absence of pathological complete response after neoadjuvant therapy (pCR). BML did not enter the final regression model. The pCR was the most prominent independent protective covariable (HR: 0.54; *p* = 0.04). See Table 3.

#### 3.1.4. Pathological Response to Therapy

The current study investigated the association of the BML with the pathological response to neoadjuvant chemoradiotherapy. Patients with no residual disease in the surgical specimen were classified as pathological complete response (pCR). The unique variable associated with pCR was squamous cell carcinoma histology. BML was not significantly associated with pCR. See Table 4.

### 3.2. Systematic Review

**Figure 5:**
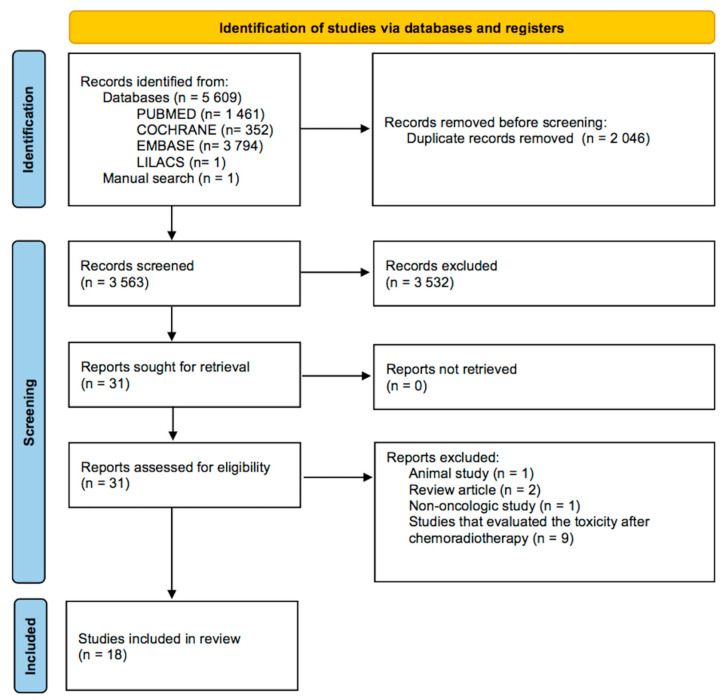
PRISMA’s selection flow diagram.

The initial search yielded 5609 articles. After applying eligibility criteria, 18 were included in the analysis [24,25,26,27,28,29,30,31,32,33,34,35,36,37,38,39,40,41] (Figure 5). All studies were observational. The baseline characteristics of the included studies can be seen in Table 5.

#### 3.2.1. Bone Marrow 18-F-FDG Uptake and Pretreatment Clinical Stage

The association of the bone marrow 18F-FDG uptake and the pretreatment clinical stage was investigated. Six studies were included for this endpoint.

Lee et al. [30,31,32] evaluated colorectal, head and neck, and breast cancer in three papers. The T3/T4 stages of bone marrow SUV (BM SUV) for colorectal cancer were significantly higher than those with T1/T2 stages. Besides, M1 patients had BM SUV higher than those with M0 stage. No difference was found for the N stage. For head and neck cancer, the T, N, and M stages influenced the value of the BM SUV. The BM SUV and the bone marrow-to-liver ratio SUV (BML) were significantly associated with the T stage for breast cancer. In contrast, BM SUV and BML were not associated with tumor size, N stage, and histologic grade.

Two studies evaluated lung cancer. Li et al. [36] investigated T1/T2 adenocarcinoma and Prévost et al. [39] investigated all non-small cell lung cancer (majoritarian adenocarcinoma). Bone marrow hypermetabolism was not associated with cStage.

#### 3.2.2. Serum Laboratory Parameters

The correlation between bone marrow metabolism and serum laboratory parameters obtained before the treatment was explored in 16 studies. Most of the laboratory tests were inflammatory parameters, such as the C-reactive protein (CRP) test, neutrophil-to-lymphocyte ratio (NLR), platelet-to-lymphocyte ratio (PLR), and white blood cell (WBC) counts.

There was high inter-study variability in the five studies that researched lung cancer [25,28,29,36,39]. Li et al. [36] evaluated T1/2N0M0 lung adenocarcinoma. Patients with high BML values had higher levels of WBC and NLR. Prévost et al. [39] found a positive correlation between bone marrow hypermetabolism after 18F-FDG PET and WBC and platelet counts and a negative correlation with the arterial partial pressure of oxygen and hemoglobin.

Lee et al. [25,28,29] evaluated three different lung cancer sets. In the set of non-small cell lung cancer treated with curative surgical resection, the authors found a positive correlation between the FDG uptake of bone marrow on PET/CT with serum CRP, NLR, and WBC count and a negative correlation with albumin level. In contrast, no significant correlation was found for hemoglobin. In the non-small cell lung cancer set treated with chemoradiotherapy, a correlation was found for WBC, CRP, and albumin but not for hemoglobin, NLR, and PLR. Finally, the authors found significant positive correlations with WBC and CRP for small cell lung cancer, but no significant association was found for NLR, hemoglobin, and platelet count.

For pleural mesothelioma [38], there was a statistically significant correlation between BML and platelet and leukocyte count.

The studies evaluated the correlation between bone marrow 18F-FDG uptake and gynecological cancer [27,40,41]. Lee et al. and Seban et al. included only cervical cancer, and no consistent correlation was found for hemoglobin, WBC, neutrophils, platelet count, and albumin. Shimura et al. included cervical, endometrial, and ovarian cancer. In their study, bone marrow 18F-FDG uptake correlated with hematological parameters, mainly the neutrophil count.

Lee et al. evaluated colorectal cancer in two papers [30,34]. Bone marrow 18F-FDG uptake was significantly positively correlated with WBC, CRP, NLR, and PLR. There was no significant correlation between BM SUV and hemoglobin.

For head and neck cancer [31], both BM SUV and BML showed significant positive correlations with CRP, NLR, and PLR. In contrast, the WBC and hemoglobin had no significant correlation with bone marrow metabolism.

In breast cancer [32], BM SUV and BML exhibited significant positive correlations with white blood cell counts, NLR, and PLR. There were no significant correlations of BM SUV or BML with serum hemoglobin.

For pancreatic cancer [33], the bone marrow metabolism showed a low correlation coefficient with NLR (correlation coefficient, 0.274) and PLR (correlation coefficient, 0.245). However, both correlations were considered significant (*p* < 0.05).

Inoue et al. [24] included lung, esophageal, head and neck, colon, and pancreas neoplasms. There were positive correlations between the bone marrow 18F-FDG uptake and WBC count, CRP, and red blood count.

#### 3.2.3. Survival Analysis

Eighteen studies investigated the association between bone marrow FDG uptake and disease-free survival (DFS), progression-free survival (PFS), or overall survival (OS) in solid neoplasms. The summary of the findings is presented in Table 6.

Lee et al. found that BML was independently associated with non-small cell lung carcinoma DFS but not for OS using multivariate Cox regression analysis [25,28]. Similarly, Li et al. [36] demonstrated a significant association between BML and DFS in lung adenocarcinoma. Prévost et al. [39] assessed both BML and BM SUV and identified associations with OS in non-small cell lung carcinoma. Mattonen et al. [37] noted that adding bone marrow PET/CT features improved the accuracy of predicting recurrence or progression.

For small cell lung carcinoma, Lee et al. [29] pointed out that BML was independently associated with PFS and OS.

In Ozmen et al.’s study [38], BML did not enter the final model of Cox regression analysis for predicting overall survival in pleural mesothelioma. However, the authors also evaluated a visual score of bone marrow 18F-FDG uptake. In multivariate analysis, patients with increased visual uptake in all the sites pelvis, lumbar spine, rib, humerus, and proximal femur had a significantly higher hazard for death (*p* = 0.042; HR: 3.82).

For breast cancer, BML was a significant predictor of DFS and distant recurrence rates [32].

Multivariate analysis revealed that BML (but not BM SUV) was an independent prognostic factor for PFS and OS in gastric cancer [26].

For colorectal cancer, bone marrow 18F-FDG uptake was an independent prognostic variable for DFS [29] and OS [34].

In pancreatic neoplasms, BM SUV was associated with OS [33].

Three studies [27,40,41] investigated the association of survival with bone marrow FDG uptake in gynecological cancers. In these studies, BM SUV could not predict OS or DFS [22,36] but could predict pelvic recurrence-free survival [40]. The BML was associated with DFS and distant recurrence-free survival [34], and the bone marrow-to-aorta SUV ratio could predict PFS [41].

Lee et al. [30] also evaluated head and neck squamous cell carcinoma, and the BML was able to predict PFS but was not able to predict distant failure-free survival. In this study, the BM SUV had no prognostic value.

Another study assessed pediatric neuroblastoma [35]. The authors classified the bone marrow 18F-FDG uptake with a visual score. The bone marrow uptake pattern was significantly associated with overall survival (HR: 0.032; *p*: 0.021), but not with recurrence free-survival (HR: 0.085; *p*: 0.062) in the multivariate analysis.

#### 3.2.4. Other Outcomes

Inoue et al. [34] compared benign and malignant conditions. The authors found that malignancy neoplasms had significantly higher BM SUV and BML values.

Shimura et al. [41] showed that patients with high bone marrow 18F-FDG uptake have increased levels of tumor-derived granulocyte colony-stimulating factor, CD33+ cells, and decreased CD8+ cells. The authors hypothesized that these immunological findings could underlie the mechanism by which bone marrow 18F-FDG uptake is associated with poor prognosis.

Lee et al. [32] found no association between BM SUV nor BML and the estrogen receptor, human epidermal growth factor receptor 2, progesterone receptor, and Ki67 expression in breast cancer.

### 3.3. Risk of Bias and Certainty Assessment

The included studies were evaluated for risk of bias by the Robins-I tool (Table 7). No major concerns were identified. However, the overall certainty assessment was very low for all outcomes. The factors that contributed to the low quality of the evidence were the absence of trials (only observational studies were included), the inter-study clinical variability, and the risk of publication bias (Table 8).

## 4. Discussion

The results of the present study showed that bone marrow hypermetabolism as measured by 18F-FDG uptake in PET/CT exams might depict systemic immunologic changes in cancer patients that could impact their prognosis.

18F-FDG bone marrow uptake in PET/CT has been routinely used in clinical practice for estimating the prognosis of hematological diseases, such as leukemia, lymphoma, and myeloma [3]. 18F-FDG bone marrow uptake is used to assess bone marrow involvement and treatment response [3].

Besides, PET/CT is routinely applied for staging certain solid neoplasms [42]. However, most clinicians only use PET/CT to assess SUV and volumetric parameters of the primary and lymph nodal disease and to define M-status [43,44]. In this sense, measuring bone marrow 18F-FDG uptake would not add any additional costs and, theoretically, could improve prognostication.

In contrast to hematological neoplasms, solid tumor bone marrow 18F-FDG uptake probably has different prognosis mechanisms. While in hematological diseases, PET/CT is used to detect the extent of the disease, in solid tumors, high bone marrow 18F-FDG uptake levels likely depict a change in the immune system. Increased bone marrow 18F-FDG-uptake accompanies the immunosuppressive cancer microenvironment, represented by increased CD33+ cells and decreased CD8+ T cells [41]. This immunosuppression mediated by granulocyte colony-stimulating factor (G-CSF) may account for the poor outcomes of patients with high bone marrow 18F-FDG-uptake [41].

However, there was high inter-study variability in the results, and the exact prognostic value of bone marrow hypermetabolism in solid tumors remains obscure. Several prognostic covariates may influence the short- and long-term outcomes in solid tumors. In esophageal cancer, the pathological response to neoadjuvant therapy is the major prognostic factor [45], and low effect size covariates may be undetected in regression models. The present cohort analysis did not show any association between pathological responses and bone marrow 18F-FDG uptake.

This study has some limitations. The studies included in this systematic review involved several different neoplasms and different therapeutic courses, which led to high inter-study clinical heterogeneity and impacted the certainty assessment. In fact, the high clinical heterogeneity rendered performing a meta-analysis impossible, and consequently, only qualitative synthesis was applied. Another issue is that one single research team from Korea was responsible for 10 out of the 18 studies included. The same authors applied a similar methodology for several different neoplasms at the same institution. Additional independent studies are needed with less clinical inter-study variability that allow for the quantitative synthesis of a systematic review.

## 5. Conclusions

18F-FDG bone marrow uptake in PET/CT has prognostic value in several solid neoplasms, including lung, gastric, colorectal, head and neck, breast, pancreas, and gynecological cancers. However, future studies are still needed to define the role of bone marrow role in cancer prognostication.

## Figures and Tables

**Table 1:** Baseline characteristics of the cohort study. Data are presented as absolute count and percentage (%). SCC: Squamous cell carcinoma; BML: Bone marrow-to-liver SUV ratio.

		Count	%
Sex	Male	83	73
	Female	30	27
Age	>65 years old	45	40
	≤65 years old	68	60
Histology	SCC	78	69
	Adenocarcinoma	35	31
Pretreatment cStage	I/II	32	28
	III/IV	81	62
BML	>1.152	56	50
	≤1.152	57	50

**Table 2:** Correlation analysis of the bone marrow-to-liver SUV ratio (BML) with serum laboratory tests. NLR: neutrophil-to-lymphocyte ratio; PLR: platelet-to-lymphocyte ratio.

	Spearman’s Rho	Prob > |t|
Albumin	−0.14	0.25
Hemoglobin	−0.17	0.08
NLR	0.13	0.19
PLR	0.11	0.26

**Table 3:** Cox regression. Univariate and multivariate analysis for disease-free survival. SCC: Squamous cell carcinoma; SE: Standard error; bone marrow-to-liver SUV ratio (BML).

Disease-Free Survival	Univariate			Multivariate		
	HR	SE	*p* > |z|	HR	SE	*p* > |z|
Sex (Male)	0.93	0.28	0.81			
Age (>65 years old)	1.99	0.51	<0.01	1.82	0.47	0.02
Histology (SCC)	0.42	0.11	<0.01	0.67	0.21	0.21
cStage (I/II)	1.94	0.59	0.03	1.36	0.47	0.37
Pathological complete response	0.44	0.12	<0.01	0.54	0.16	0.04
BML (>1.15)	1.34	0.34	0.26			

**Table 4:** Logistic regression. Univariate and multivariate analysis for pathological response to neoadjuvant chemoradiotherapy. SCC: Squamous cell carcinoma; SE: Standard error; bone marrow-to-liver SUV ratio (BML).

Pathological Complete Response	Univariate			Multivariate		
	OR	SE	*p* > |z|	OR	SE	*p* > |z|
Sex (Male)	1.34	0.6	0.44			
Age (>65 years old)	0.87	0.33	0.7			
Histology (SCC)	18	12	<0.01	18	12	<0.01
cStage (I/II)	0.55	0.23	0.16			
BML (>1.15)	0.81	0.31	0.58			

**Table 5:** Baseline characteristics of the included studies selected in systematic search. BML: Bone marrow-to-liver ratio; BM: Bone marrow; BMAo: Bone marrow-to-aorta ratio; OS: Overall survival; PFR/DFS: Progression-free survival or disease-free survival; GLCM: Grey Level Co-occurrence Matrix Pelvic; PRFS: Recurrence-free survival; EPRFS: Extra-pelvic recurrence free survival; pCR: pathological complete response.

Author Year	Design	Neoplasm	Site of Uptake Measurement	PET/CT Evaluation	N	Mean Age (Years)	Mean Follow-Up (Months)	Therapy	Outcomes
Inoue 2009	Cohort	Lung, esophageal, head and neck, colon, pancreas	Thoracic (T10-12) and lumbar (L2-4) vertebrae	BML SUV	32	62	at least 6	Uninformed	Laboratory parameters, comparison malignant vs. benign
Lee 2016	Cohort	Lung cancer	Thoracic (T11-12) and lumbar (L3-5) vertebrae	BML SUV and BM SUV	110	65	22	Surgical resection	OS, PFS/DFS, laboratory parameters
Lee 2017.1	Cohort	Gastric cancer	Thoracic (T10-12) and lumbar (L3-5) vertebrae	BML SUV and BM SUV	309	60	34	Surgical resection	OS, PFS/DFS
Lee 2017.2	Cohort	Cervical cancer	Thoracic (T11-12) and lumbar (L3-5) vertebrae	BML SUV and BM SUV	145	52	26	Chemoradiotherapy or surgical resection	PFS, DRFS, laboratory parameters
Lee 2017.3	Cohort	Lung cancer	Thoracic (T11-12) and lumbar (L3-5) vertebrae	BML SUV and BM SUV	106	74	19	Chemoradiotherapy	OS, PFS/DFS, laboratory parameters
Lee 2018.1	Cohort	Lung cancer	Thoracic and lumbar vertebrae	BM SUV	70	68	11	Chemotherapy, radiotherapy, or surgical resection	OS, PFS/DFS, laboratory parameters
Lee 2018.2	Cohort	Colorectal cancer	Thoracic (T10-12) and lumbar (L3-5) vertebrae	BM SUV	226	66	32	Surgical resection	PFS/DFS, laboratory parameters, cStage
Lee 2019	Cohort	Head and neck	Thoracic and lumbar vertebrae	BML SUV and BM SUV	157	61	26	Chemotherapy, radiotherapy or surgical resection	PFS/DFS, DRRS, cStage, laboratory parameters
Lee 2020	Cohort	Breast cancer	Thoracic and lumbar vertebrae	BML SUV and BM SUV	345	51	49	Surgical resection (with or without neoadjuvant therapy)	PFS/DFS, DRRS, cStage, laboratory parameters
Lee 2021.1	Cohort	Pancreas cancer	Thoracic and lumbar vertebrae	BML SUV and BM SUV	65	66	Uninformed	Chemotherapy, radiotherapy, or surgical resection	OS
Lee 2021.2	Cohort	Colorectal cancer	Thoracic and lumbar vertebrae	SLR SUV and BML SUV	411	Uninformed	91	Surgical resection (with or without chemotherapy)	OS, laboratory parameters
Li 2018	Cohort	Neuroblastoma	Uninformed	BM SUV	47	2	24	Surgical resection (with or without chemotherapy or radiotherapy)	OS, PFS/DFS
Li 2020	Cohort	Lung cancer	Thoracic and lumbar vertebrae	BML SUV and BM SUV	195	63	4 to 65	Surgical resection	PFS/DFS, laboratory parameters, cStage
Mattonen 2019	Cohort	Lung cancer	Lumbar vertebrae (L3-L5)	GLCM	227	70	41	Surgical resection	PFS/DFS
Ozmen 2016	Cohort	Pleural mesothelioma	Lumbar vertebrae (L3-L5)	BML SUV and BM SUV	51	56	28 to 56	Surgical resection, chemotherapy, or palliation therapy	OS, laboratory parameters
Prévost 2016	Cohort	Lung cancer	Lumbar vertebrae (L3-L5)	BML SUV and BM SUV	120	68	18	Chemotherapy, radiotherapy, or surgical resection	OS, laboratory parameters, cStage
Seban 2019	Cohort	Cervical cancer	Thoracic (T12) and lumbar (L3-5) vertebrae	BM SUV	116	47	75	Chemoradiotherapy, brachytherapy	OS, PRFS, EPRFS, laboratory parameters
Shimura 2021	Cohort	Gynecological cancer	Thoracic (T8-12) vertebrae	BMAo and BM SUV	559	56	48	Surgical resection	PFS/DFS
Current study 2022	Cohort	Esophageal cancer	Lumbar vertebrae (L3-L5)	BML SUV and BM SUV	113	61	25	Trimodal	PFR/DFS, pCR, cStage, laboratory parameters

**Table 6:** Summary of the survival outcomes in the included studies. * Survival analyses were expressed as hazard ratio from multivariate models of FDG bone marrow uptake association with survival. Gray hatched outcomes show statistical significant association. OS: Overall survival; PFR: Progression-free survival; DFS: Disease-free survival. n.s.: Non significant association.

Author Year	Neoplasm	Survival Analysis *
Lee 2016	Lung cancer	DFS: 2.41
		OS: 2.15 (n.s.)
Lee 2017.1	Gastric cancer	DFS: 8.25
		OS: 20.69
Lee 2017.2	Cervical cancer	PFS: 2.32
Lee 2017.3	Lung cancer	PFS: 14.44
		OS: 1.24 (n.s.)
Lee 2018.1	Lung cancer	PFS: 2.28
		OS: 1.47 (n.s.)
Lee 2018.2	Colorectal cancer	DFS: 2.94
Lee 2019	Head and neck	PFS: 1.96
Lee 2020	Breast cancer	DFS: 16.38
Lee 2021.1	Pancreas cancer	OS: 4.3
Lee 2021.2	Colorectal cancer	OS: 5.28
Li 2018	Neuroblastoma	RFS: 0.085 (n.s.)
		OS: 0.032
Li 2020	Lung cancer	RFS: 5.09 (n.s.)
Mattonen 2019	Lung cancer	RFS: 1.62
Ozmen 2016	Pleural mesothelioma	OS: 3.82
Prévost 2016	Lung cancer	OS: 1.6
Seban 2019	Cervical cancer	OS: 2.7
Shimura 2021	Gynecological cancer	PFS: 3.07
Current study 2022	Esophageal cancer	DFS: 1.34 (n.s.)

**Table 7:** The ROBINS-I tool for assessment for non-randomized studies risk of bias, based on the domains confounding, selection, classification of interventions, deviation from intended intervention, missing data, measurement of endpoints, and reporting bias. Each domain was scored as low, moderate, or serious.

Author	1. Bias due to Confounding	2. Bias in Selection of Participants into the Study	3. Bias in Classification of Interventions	4. Bias due to Deviations from Intended Interventions	5. Bias due to Missing Data	6. Bias in Measurement of Outcomes	7. Bias in Selection of the Reported Results	8. Overall Bias
Inoue 2009	Low	Low	Low	Low	Serious	Low	Moderate	Low
Lee 2016	Low	Low	Low	Low	Moderate	Low	Moderate	Low
Lee 2017.1	Low	Low	Low	Low	Moderate	Low	Moderate	Low
Lee 2017.2	Low	Low	Low	Low	Moderate	Low	Moderate	Low
Lee 2017.3	Low	Low	Low	Low	Moderate	Low	Moderate	Low
Lee 2018.1	Low	Low	Low	Low	Moderate	Low	Moderate	Low
Lee 2018.2	Low	Low	Low	Low	Moderate	Low	Moderate	Low
Lee 2019	Low	Low	Low	Low	Moderate	Low	Moderate	Low
Lee 2020	Low	Low	Low	Low	Moderate	Low	Moderate	Low
Lee 2021.1	Low	Low	Low	Low	Moderate	Low	Moderate	Low
Lee 2021.2	Low	Low	Low	Low	Moderate	Low	Moderate	Low
Li 2018	Low	Low	Low	Low	Moderate	Low	Moderate	Low
Li 2020	Low	Low	Low	Low	Moderate	Low	Moderate	Low
Mattonen 2019	Low	Low	Low	Low	Moderate	Low	Moderate	Low
Ozmen 2016	Low	Low	Low	Low	Moderate	Low	Moderate	Low
Prévost 2016	Low	Low	Low	Low	Moderate	Low	Moderate	Low
Seban 2019	Low	Low	Low	Low	Moderate	Low	Moderate	Low
Shimura 2021	Low	Low	Low	Low	Moderate	Low	Moderate	Low
Current study 2022	Low	Low	Low	Low	Low	Low	Low	Low

**Table 8:** Grading of Recommendations, Assessment, Development, and Evaluations-GRADE was used for the certainty assessment of the evidence and the strength of recommendations. This tool is based on risk of bias, inconsistency, indirectness, and imprecision. ^a^. High clinical heterogeneity; ^b^. Risk of publication bias.

Certainty Assessment						
Studies	Risk of Bias	Inconsistency	Indirectness	Imprecision	Publication Bias	Overall Certainty of Evidence
**Clinical stage**						
6 observational studies	not serious	very serious ^a^	not serious	not serious	publication bias strongly suspected ^b^	⨁◯◯◯ Very low
**Serum laboratory parameters**						
16 observational studies	not serious	very serious ^a^	not serious	not serious	publication bias strongly suspected ^b^	⨁◯◯◯ Very low
**Survival**						
18 observational studies	not serious	very serious ^a^	not serious	not serious	publication bias strongly suspected ^b^	⨁◯◯◯ Very low

## Data Availability

Data available on request from the authors.
